# Collodion in Erysipelas

**Published:** 1851-03

**Authors:** 


### ARTICLE XIV.

### *Collodion in Erysipelas. A Singular Coincidence.*

In the December number of this Journal, we noticed the successful treatment of a case of Erysipelas by the application of Collodion, the report of which, with that of four other cases was published in the Lancet of January. We have since that time employed this agent in very many instances, and with the most prompt and flattering results.

In the Eclectic Department of this Number, our readers will find a paper from the London Lancet of December, reporting the use of Collodion in the same affection by the well known Surgeon, Mr. Luke. It is somewhat singular, that probably about the same time, and with the same intentions, the Collodion was employed by us both. And the coincidence is the more remarkable, that the great similarity of expression and pathological deductions, might lead some to suppose that we were in *telegraphic* communication with Mr. Pete, the Reporter of the London Hospital.—*Northern Lancet for February*, 1851.

We quote the above to give our readers another illustration of the intensity with which the Medical public, in this country, keep their eyes fixed upon the East; scarcely surpassed by the superstitious devotions of the Arabs towards Mecca.

By reference to the files of our Journal it will be found that just one year before the publication of the article above referred to, in the London Lancet, Dr. J. W. Freer, (now Demonstrator of Anatomy in Rush Medical College) published the result of a number of cases of Erysipelas, which had been treated by the Application of Collodion; his use of it commencing eight months previously to the publication. His reasoning was the same as Mr. Luke's. It is a remarkable coincidence that Dr. John Snow of London, reported the same treatment in the *London Lancet, April 29th, 1850; just four months after the article of Dr. Freer was given to the public in our Journal. We are not apprized of the precise length of time it takes our Journals to cross the Atlantic, but should think it ought not to be over four months. E.

*See Braithwaite's Retrospect of July 1850.

